# Diastolic orthostatic hypertension and cardiovascular prognosis in type 2 diabetes: a prospective cohort study

**DOI:** 10.1186/s12933-016-0399-0

**Published:** 2016-06-02

**Authors:** Magnus Wijkman, Toste Länne, Carl Johan Östgren, Fredrik H. Nystrom

**Affiliations:** Department of Internal Medicine and Department of Medical and Health Sciences, Linköping University, Norrköping, Sweden; Department of Medical and Health Sciences, Linköping University, Linköping, Sweden; Department of Internal Medicine, Vrinnevi Hospital, Gamla Övägen 25, 603 79 Norrköping, Sweden

**Keywords:** Arterial stiffness, Blood pressure, Cardiovascular risk, Carotid intima-media thickness, Events, Orthostatic hypotension, Type 2 diabetes mellitus

## Abstract

**Background:**

In patients with type 2 diabetes, the prognostic impact of an orthostatic rise in blood pressure is not known. Therefore, the aim of this study was to determine the prognostic implications of the diastolic orthostatic blood pressure response in a cohort of patients with type 2 diabetes. We also evaluated associations between different orthostatic blood pressure responses and markers of subclinical cardiovascular organ damage.

**Methods:**

Office blood pressures were measured in the sitting and in the standing position in 749 patients with type 2 diabetes who participated in the CARDIPP study (Cardiovascular Risk factors in Patients with Diabetes—a Prospective study in Primary care). Diastolic orthostatic hypertension was defined as a rise of diastolic blood pressure ≥10 mmHg and diastolic orthostatic hypotension was defined as a drop of diastolic blood pressure ≥10 mmHg. Recruitment took place between the years 2005–2008, and patients were followed until any of the primary outcome events (cardiovascular death or hospitalization for either myocardial infarction or stroke) occurred or until December 31st, 2014. Measurements of aortic pulse wave velocity and of carotid intima-media thickness were performed at base-line.

**Results:**

Diastolic orthostatic hypertension was found in 140 patients (18.7 %) and was associated with significantly lower risk of cardiovascular events (crude hazard ratio compared with patients with normal systolic and diastolic orthostatic blood pressure response: 0.450, 95 % C.I. 0.206–0.987, *P* = 0.046). Diastolic orthostatic hypotension was found in 31 patients (4.1 %) and was associated with higher values for aortic pulse wave velocity and carotid intima-media thickness, compared with patients with normal systolic and diastolic orthostatic blood pressure response.

**Conclusions:**

Diastolic orthostatic hypertension is common in patients with type 2 diabetes, and may be a novel marker for decreased cardiovascular risk in these patients.

## Background

In patients with type 2 diabetes, orthostatic hypotension is a common clinical finding [1], which is associated with an increased risk for cardiovascular mortality [2]. Orthostatic hypotension is also an established marker for cardiovascular risk in patients with hypertension [3, 4], in the elderly [5–8] and in the general population [9–12]. Considerably less is known, however, about the prevalence and clinical impact of the reverse phenomenon, orthostatic hypertension, which implies an exaggerated rise in blood pressure when assuming an upright position [13, 14]. Orthostatic hypertension has been associated with increased prevalence of silent cerebrovascular lesions in the elderly [15], and with increased risk for incident lacunar strokes in the general population [16]. In patients with type 2 diabetes, an increased prevalence of orthostatic hypertension has been described [17], and orthostatic hypertension has been associated with markers of neuropathy [17] and with altered indices of left atrial volume [18] but prospective studies concerning the predictive value of orthostatic hypertension are lacking. Therefore, we measured the diastolic and systolic blood pressure responses to rising from the sitting to the standing position in a Swedish cohort of middle-aged patients with type 2 diabetes, and analyzed their influence on cardiovascular prognosis. We also explored differences in the extent of subclinical cardiovascular organ damage, measured as aortic pulse wave velocity (PWV) and carotid intima-media thickness (IMT), between patients with differing orthostatic blood pressure responses.

## Methods

### Population

As described previously [19], CARDIPP (Cardiovascular Risk Factors in Patients with Diabetes—a Prospective Study in Primary Care) is an observational prospective cohort study with the general aim of exploring the impact of cardiovascular risk factors in patients with type 2 diabetes (Clinical Trials.gov number NCT 01049737). Between the years 2005–2008, 761 patients with type 2 diabetes were recruited by nurses specially trained in diabetes care at 22 different primary health care centers in the counties of Östergötland and Jönköping in Sweden. Inclusion criteria were: type 2 diabetes treated in primary care; age 55–65 years; and willingness to participate in the study. The only exclusion criterion was known severe physical or mental disease with short life expectancy. From the total CARDIPP cohort of 761 patients, we excluded for the purpose of this analysis 12 patients for which base-line data were missing for systolic or diastolic blood pressure in either the sitting or the standing position. This yielded a study sample of 749 participants.

### Outcomes

The primary endpoint was a composite endpoint of the first non-fatal or fatal event of hospitalization for acute myocardial infarction (ICD-10 code I21), or stroke (ICD-10 codes I60, I61, I63.0–I63.5, I63.8–I63.9, I64) or cardiovascular mortality (ICD-10 codes I00–99). Patients were followed until a primary outcome event occurred, or until December 31st, 2014, by linkage to the Swedish Cause of Death and Inpatient registries (The National Board of Health and Welfare, Stockholm, Sweden) using the national personal identification number of each individual patient, thus ensuring coverage of the entire population.

### Sitting and standing blood pressure measurements

Office blood pressure was measured by specially trained nurses [20]. Measurements were made manually with the auscultatory method, with appropriately sized cuffs, following a 5 minute wait, with the patient sitting comfortably in a relaxed position with the arm supported at heart level. Three sitting measurements were made, with 1 minute between each measurement. Thereafter, the patient was asked to rise, and one additional measurement was made when the patient had been standing for 2 minutes. Blood pressures were determined to the nearest 2 mmHg. The blood pressure value obtained at the third sitting measurement is referred to as the sitting blood pressure in this report. The orthostatic blood pressure response was calculated as the difference between the third sitting measurement and the standing measurement. The cut-off for abnormal systolic orthostatic blood pressure responses was ≥20 mmHg, as has been proposed for both hypotensive [21] and hypertensive [13] orthostatic blood pressure reactions. Thus, systolic orthostatic hypertension was defined as a rise of systolic blood pressure ≥20 mmHg, with or without an accompanying diastolic blood pressure rise, and systolic orthostatic hypotension was defined as a drop of systolic blood pressure ≥20 mmHg, with or without an accompanying diastolic blood pressure drop. For diastolic orthostatic hypotensive reactions the cut-off was ≥10 mmHg [21] and we applied the same numerical threshold for diastolic orthostatic hypertension, since no consensus criteria exist for this entity [13]. Accordingly, diastolic orthostatic hypertension was defined as a rise of diastolic blood pressure ≥10 mmHg, with or without an accompanying systolic blood pressure rise, and diastolic orthostatic hypotension was defined as a drop of diastolic blood pressure ≥10 mmHg, with or without an accompanying drop of systolic blood pressure. Thus, a normal systolic orthostatic blood pressure response was defined as a change of systolic blood pressure within the range of −19 to +19 mmHg, and a normal diastolic orthostatic blood pressure response was defined as a change of diastolic blood pressure within the range of −9 to +9 mmHg.

### Aortic pulse wave velocity measurements

To measure aortic PWV, electrocardiogram-gated pulse wave analyses of the carotid and femoral arteries were performed with a Millar pressure tonometer and the SphygmoCor system (Model MM3, AtCor Medical, Sydney, Australia). The pulse wave transit time was calculated by subtracting the time between the ECG R-wave and the arrival of the pulse wave to the carotid measurement site from the time between the ECG R-wave and the arrival of the pulse wave to the femoral measurement site. The surface distance was defined as the distance between the suprasternal notch and the femoral measurement site, subtracted by the distance between the suprasternal notch and the carotid measurement site. The PWV was calculated by dividing the surface distance with the pulse wave transit time. Applanation tonometry measurements were performed in duplicate, and reported as means of the two measurements. Measures of PWV were successfully obtained in 692 patients at base-line.

### Carotid ultrasonography

Carotid IMT was measured in B-mode, using Philips ATL HDI 5000 (Philips Ultrasound, Seattle, USA) with a 4–7 MHz linear transducer [22]. Three consecutive longitudinal images, frozen in diastole, were analyzed with software for off-line measurement of intima-media thickness (Artery Measurement System II; Image and Data Analysis, Gothenburg, Sweden). A section of 10 mm 1–2 cm proximal to the carotid bulb was measured manually by tracing a cursor along the echo wedges. A mean value from the right and left carotid arteries was calculated. Measures of carotid IMT were successfully obtained in 729 patients at base-line.

### Laboratory analyses

Blood samples were drawn in the morning following a 10 h over-night fast. The Swedish Mono-S HPLC standard was used for HbA1c analyses [23], but the values were subsequently converted to the International Federation of Clinical Chemistry and Laboratory Medicine (IFCC) units (mmol/mol). Creatinine-based values of estimated glomerular filtration rate (eGFR) were calculated according to the Modification of Diet in Renal Disease (MDRD) study equation [24].

### Statistics

For the statistical analyses, SPSS software (IBM SPSS Statistics 23, Chicago, IL, USA) was used. In the main comparisons, the reference group was defined as patients who had a normal systolic and diastolic orthostatic blood pressure response. Differences of base-line characteristics were tested for statistical significance with two-sided independent *t* test, Chi square test or, where appropriate, Fisher's exact test. With the use of Cox regression models, the associations between the time to a first endpoint event and the presence of diastolic or systolic orthostatic hypertension or hypotension, were calculated as the hazard ratio (HR) for each group with a corresponding 95 % confidence interval (C.I.). Crude HRs were first calculated, and if they were significant, adjusted HRs were then calculated by using multivariate Cox regression models which adjusted for traditional cardiovascular risk factors. The first multivariate model adjusted for age, sex and sitting systolic blood pressure, and the second multivariate model adjusted additionally for smoking status, low density lipoprotein (LDL) cholesterol, body mass index and use of any antihypertensive medication. If the crude hazard ratios were not statistically significant, no further adjustments were made. Statistical significance was defined as *P* < 0.05. Since the purpose of the study was to present adjusted HRs, and not to screen for independent predictors, no adjustments for multiple testing were performed.

### Ethics

All participants gave written informed consent. The merging of study data with other registries was approved by the National Board of Health and Welfare and by the Swedish Data Inspection Board. The study was approved by the Regional Ethical Review Board in Linköping, Sweden. The study protocol followed the principles expressed in the Declaration of Helsinki.

## Results

### Population characteristics

The study population consisted of 257 women (34.3 %) and 492 men. The majority (88.5 %) were born in Sweden. The most common anti-diabetic treatment strategy was oral anti-diabetics (OAD) and/or non-insulin injectables (NNI) which were used by 40.9 percent of the participants, followed by life-style advice only (28.2 %), combined use of insulin with OAD/NNI (18.0 %) and insulin only (13.0 %). There were 462 participants (61.7 %) who reported a previous diagnosis of hypertension, 67 participants (8.9 %) who reported having had a myocardial infarction and 18 participants (2.4 %) who reported having had a stroke. Background characteristics are presented according to the orthostatic blood pressure reaction (Table 1). During a median follow-up time of 7.8 years (range: 38–3537 days), there were 75 end-point events (42 events related to ischemic heart disease including 37 myocardial infarctions, 27 events related to ischemic stroke, four events related to intracerebral hemorrhage, one event related to subdural bleeding and one event related to congestive heart failure). Fifty-eight events occurred among the 534 patients with normal systolic and diastolic orthostatic blood pressure responses (10.9 %), five events occurred among the 45 patients with systolic orthostatic hypertension (11.1 %), two events occurred among the 24 patients with systolic orthostatic hypotension (8.3 %), seven events occurred among the 140 patients with diastolic orthostatic hypertension (5.0 %) and six events occurred among the 31 patients with diastolic orthostatic hypotension (19.4 %).

**Table 1:** Clinical characteristics and medications at base-line in 749 patients with type 2 diabetes, stratified according to their orthostatic blood pressure reactions

	Normal orthostatic blood pressure response (n = 534)	Systolic orthostatic hypertension (*n* = 45)	Systolic orthostatic hypotension (n = 24)	Diastolic orthostatic hypertension (n = 140)	Diastolic orthostatic hypotension (*n* = 31)
Age (years)	60.7 ± 3.0	60.4 ± 3.5	60.8 ± 2.6	60.4 ± 3.1	61.5 ± 3.3
Women, *n* (%)	183 (34.3 %)	18 (40 %)	7 (29.2 %)	47 (33.6 %)	11 (35.5 %)
Diabetes duration (years)	7.0 ± 5.4	6.3 ± 5.4	9.2 ± 11.2	7.4 ± 7.5	8.7 ± 6.0
Previous MI, *n* (%)	52 (9.8 %)	2 (4.4 %)	2 (8.3 %)	10 (7.1 %)	2 (6.5 %)
Previous stroke, *n* (%)	10 (1.9 %)	3 (6.8 %)	0 (0.0 %)	6 (4.3 %)	1 (3.2 %)
BMI (kg/m^2^)	30.1 ± 4.7	31.8 ± 5.1*	29.7 ± 4.9	30.0 ± 4.9	30.0 ± 4.5
HbA1c, Mono S (%)	6.1 ± 1.2	5.9 ± 1.0	6.2 ± 0.9	6.1 ± 1.0	6.2 ± 0.9
HbA1c, IFCC (mmol/mol)	52.9 ± 12.3	51.2 ± 10.5	54.2 ± 9.6	52.8 ± 10.5	54.5 ± 9.7
eGFR (ml/min/1.73 m^2^)	74.1 ± 16.1	80.8 ± 21.4	76.4 ± 23.7	77.5 ± 18.5	74.7 ± 17.7
Total cholesterol (mmol/l)	4.7 ± 0.9	5.0 ± 1.1	4.8 ± 1.0	4.8 ± 1.0	4.7 ± 1.1
HDL cholesterol (mmol/l)	1.3 ± 0.3	1.3 ± 0.3	1.3 ± 0.3	1.3 ± 0.4	1.3 ± 0.3
LDL cholesterol (mmol/l)	2.7 ± 0.8	2.9 ± 0.8	2.7 ± 0.8	2.8 ± 0.8	2.5 ± 0.8
Triglycerides (mmol/l)	1.8 ± 1.0	2.0 ± 1.5	1.8 ± 1.1	1.7 ± 1.1	2.0 ± 1.2
Resting heart rate (bpm)	66.7 ± 11.3	66.2 ± 11.9	68.1 ± 13.8	66.4 ± 10.7	69.7 ± 13.6
Diabetes treatment, *n* (%)					
Lifestyle only	155 (29.0 %)	19 (42.2 %)	5 (20.8 %)	35 (25.0 %)	4 (12.9 %)
OAD/NNI	217 (40.6 %)	16 (35.6 %)	11 (45.8 %)	59 (42.1 %)	15 (48.4 %)
Insulin	74 (13.9 %)	1 (2.2 %)	4 (16.7 %)	15 (10.7 %)	5 (16.1 %)
Insulin + OAD/NNI	88 (16.5 %)	9 (20.0 %)	4 (16.7 %)	31 (22.1 %)	7 (22.6 %)
Beta-blockers, *n* (%)	184 (34.4 %)	18 (40.0 %)	8 (33.3 %)	52 (37.1 %)	12 (38.7 %)
Loop diuretics, *n* (%)	42 (7.9 %)	5 (11.1 %)	2 (8.7 %)	11 (7.9 %)	1 (3.2 %)
Thiazide diuretics, *n* (%)	51 (9.6 %)	6 (13.3 %)	2 (8.7 %)	16 (11.5 %)	5 (16.1 %)
ACEI/ARB, *n* (%)	229 (42.9 %)	19 (42.2 %)	9 (39.1 %)	64 (45.7 %)	13 (41.9 %)
CCB, *n* (%)	85 (15.9 %)	6 (13.3 %)	4 (17.4 %)	15 (10.8 %)	6 (19.4 %)
Any BP medication, *n* (%)	343 (64.2 %)	28 (62.2 %)	14 (58.3 %)	100 (71.4 %)	19 (61.3 %)
Statin treatment, *n* (%)	293 (55.0 %)	20 (44.4 %)	11 (47.8 %)	76 (54.3 %)	17 (54.8 %)
Smoking status, *n* (%)					
Never smoked	172 (32.9 %)	17 (37.8 %)	6 (25.0 %)	39 (28.1 %)	9 (29.0 %)
Former smoker	256 (48.9 %)	19 (42.2 %)	12 (50.0 %)	72 (51.8 %)	14 (45.2 %)
Current smoker	95 (18.2 %)	9 (20.0 %)	6 (25.0 %)	28 (20.1 %)	8 (25.8 %)

Since 25 patients had overlapping orthostatic blood pressure reactions (for details please see text), the sum of the *n* numbers of all strata is 774

Number of patients with missing data: 45 (diabetes duration), 1 (previous myocardial infarction), 4 (previous stroke), 1 (BMI), 10 (HbA1c), 23 (eGFR), 23 (total cholesterol), 26 (HDL cholesterol), 58 (LDL cholesterol), 29 (triglycerides), 57 (aortic PWV), 21 (carotid IMT), 15 (resting heart rate), 1 (ACEI/ARB); 2 (diuretics); 2 (CCB); 2 (statin); 12 (smoking status)

*ACEI/ARB* angiotensin converting enzyme inhibitors/angiotensin receptor blockers,* BMI* body mass index,*BP* blood pressure, *bpm* beats per minute; *CCB* calcium channel blockers; *eGFR* estimated glomerular filtration rate; *HbA1c* glycosylated HemoglobinA1; *HDL* high-density lipoprotein; *OAD/NNI* oral antidiabetes drugs/non-insulin injectables; *LDL* low-density lipoprotein

* Denotes a statistically significant difference at the *P* < 0.05 level, compared with the normal group

### Orthostatic blood pressure response

**Fig. 1:**
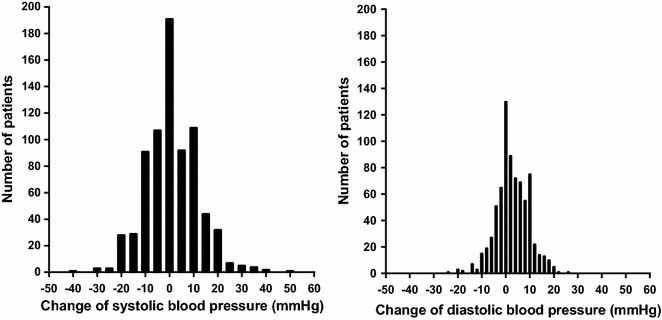
Distribution of systolic (*left panel*) and diastolic (*right panel*) orthostatic blood pressure changes, in 749 patients with type 2 diabetes

The difference between the sitting blood pressure and the standing blood pressure followed normal distribution curves (Fig. 1) for both systolic and diastolic measurements. For the systolic blood pressure change, mean (SD) was 1.1 (10.9) mmHg, median was 0 mmHg and the range was −40 to +50 mmHg (inter-quartile range −6 to +8 mmHg). For the diastolic blood pressure change, mean (SD) was 2.6 (6.8) mmHg, median was 2.0 mmHg and the range was −24 to +25 mmHg (inter-quartile range −2 to +8 mmHg). The most common orthostatic abnormality was diastolic hypertension which was found in 140 patients (18.7 %) followed by systolic hypertension which was found in 45 patients (6.0 %), diastolic hypotension which was found in 31 patients (4.1 %) and systolic hypotension which was found in 24 patients (3.2 %). There were 25 patients (3.4 %) with more than one orthostatic blood pressure abnormality: Thirteen of the patients with systolic orthostatic hypertension also had diastolic orthostatic hypertension, six of the patients with systolic orthostatic hypotension also had diastolic orthostatic hypotension, four of the patients with diastolic orthostatic hypertension also had systolic orthostatic hypotension, and two of the patients with diastolic orthostatic hypotension also had systolic orthostatic hypertension. Overall, there were 534 patients (71.3 %) who had a normal orthostatic blood pressure response, defined as a change of systolic blood pressure within the range of −19 to +19 mmHg and a change of diastolic blood pressure within the range of −9 to +9 mmHg. This group was used as the reference group in all main comparisons. Sitting and standing blood pressures are presented according to the systolic and diastolic orthostatic blood pressure reactions in Table 2.

**Table 2:** Blood pressure variables and markers of target organ damage in 749 patients with type 2 diabetes, stratified according to their orthostatic blood pressure reactions

	Normal orthostatic blood pressure response (n = 534)	Systolic orthostatic hypertension (*n* = 45)	Systolic orthostatic hypotension (*n* = 24)	Diastolic orthostatic hypertension (*n* = 140)	Diastolic orthostatic hypotension (*n* = 31)
Sitting SBP (mmHg)	135.2 ± 16.1	132.2 ± 15.8	147.0 ± 15.2^†^	136.1 ± 17.7	146.4 ± 18.0^†^
Sitting DBP (mmHg)	79.6 ± 10.0	83.6 ± 12.3*	79.7 ± 11.3	77.7 ± 10.6*	86.7 ± 9.3^†^
Standing SBP (mmHg)	134.7 ± 16.8	157.3 ± 15.4^†^	124.0 ± 16.4^†^	142.5 ± 17.7^†^	137.7 ± 22.2
Standing DBP (mmHg)	80.6 ± 10.5	88.8 ± 13.3^†^	78.4 ± 13.8	90.0 ± 10.5^†^	73.5 ± 10.3^†^
Aortic PWV (m/s)	10.2 ± 2.1	11.3 ± 2.5^†^	11.0 ± 3.0	10.6 ± 2.1	11.3 ± 2.7*
Carotid IMT (mm)	0.73 ± 0.2	0.74 ± 0.1	0.79 ± 0.3	0.75 ± 0.2	0.83 ± 0.2*

Since 25 patients had overlapping orthostatic blood pressure reactions (for details please see text), the sum of the *n* numbers of all strata is 774

Number of patients with missing data: 57 (Aortic PWV), 20 (Carotid IMT)

*DBP* diastolic blood pressure; *IMT* intima-media thickness; *PWV* pulse wave velocity; *SBP* systolic blood pressure

* Denotes a statistically significant difference at the *P* < 0.05 level

^†^Denotes a statistically significant difference at the *P* < 0.01 level, compared with the normal group

### Systolic orthostatic hypertension

In a Cox regression analysis that compared patients with systolic orthostatic hypertension (*n* = 45) with the 534 patients who had a normal systolic and diastolic orthostatic blood pressure response, systolic orthostatic hypertension was not significantly associated with the risk of the combined end-point (HR 1.095, 95 % C.I. 0.439–2.731, *P* = 0.486). Similar results were obtained when the 45 patients with systolic orthostatic hypertension were compared with the 704 patients without systolic orthostatic hypertension (HR 1.201, 95 % C.I. 0.485–2.976, *P* = 0.693) or with the 680 patients who had a normal systolic blood pressure response (HR 1.191, 95 % C.I. 0.480–2.954, *P* = 0.706). Patients with systolic blood pressure rise ≥20 mmHg had significantly (*P* = 0.001) higher PWV than patients with a normal orthostatic blood pressure response, but similar IMT (Table 2).

### Systolic orthostatic hypotension

In a Cox regression analysis that compared patients with systolic orthostatic hypotension (*n* = 24) with the 534 patients who had a normal systolic and diastolic orthostatic blood pressure response, systolic orthostatic hypotension was not significantly associated with the risk of the combined end-point (HR 0.750, 95 % C.I. 0.183–3.073, *P* = 0.690). Similar results were obtained when the 24 patients with systolic orthostatic hypotension were compared with the 725 patients without systolic orthostatic hypotension (HR 0.810, 95 % C.I. 0.199–3.301, *P* = 0.769) or with the 680 patients who had a normal systolic blood pressure response (HR 0.819, 95 % C.I. 0.201–3.340, *P* = 0.780). Patients with systolic blood pressure fall ≥ 20 mmHg had non-significantly higher PWV and IMT compared with patients with a normal orthostatic blood pressure response (Table 2).

### Diastolic orthostatic hypertension

Patients with diastolic orthostatic hypertension (*n* = 140) had a significantly lower risk of experiencing the combined end-point compared with the 534 patients who had a normal systolic and diastolic orthostatic blood pressure reaction (HR 0.450, 95 % C.I. 0.206–0.987, *P* = 0.046). Diastolic orthostatic hypertension remained significantly associated with a decreased risk for cardiovascular events following adjustment for age, sex and sitting systolic blood pressure (HR 0.452, 95 % C.I. 0.206–0.990, *P* = 0.047, cumulative event rate 65/674) as well as following additional adjustment for smoking status, LDL cholesterol, body mass index and use of any antihypertensive medication (HR 0.335, 95 % C.I. 0.133–0.839, *P* = 0.020, cumulative event rate 59/616). The results of the fully adjusted model are presented in Table 3. Patients with diastolic orthostatic hypertension did not differ significantly from patients with a normal orthostatic blood pressure response in terms of PWV and IMT (Table 2). Among the patients with diastolic orthostatic hypertension, 88 (62.9 %) had a standing diastolic blood pressure ≤90 mmHg and 72 (51.4 %) had a standing systolic blood pressure ≤140 mmHg.

**Table 3:** Adjusted hazard ratios for a composite end-point consisting of cardiovascular death or hospitalization for either myocardial infarction or stroke, based on a multivariate Cox regression model in 616 patients with type 2 diabetes

	HR	95 % C.I.	*P*
Diastolic orthostatic hypertension	0.335	0.133–0.839	0.020
Age, 1 year	1.060	0.969–1.159	0.201
Female gender	0.543	0.297–0.993	0.047
Sitting systolic blood pressure, 5 mmHg	1.097	1.015–1.186	0.019
Current smoker	1.452	0.759–2.777	0.260
LDL cholesterol, 1 mmol/l	1.057	0.754–1.482	0.748
BMI, 1 kg/m^2^	1.071	1.017–1.128	0.010
Any antihypertensive medication	1.410	0.750–2.653	0.286

Cumulative event rate: 59/616 = 9.6 %

*BMI* body mass index; *C.I.* confidence interval; *LDL* low density lipoprotein

### Diastolic orthostatic hypotension

There was a non-significant trend towards increased risk for the combined end-point when patients with diastolic orthostatic hypotension (*n* = 31) were compared with the 534 patients who had a normal systolic and diastolic blood pressure response (HR 1.804, 95 % C.I. 0.778–4.183, *P* = 0.169). The same non-significant trend was observed when patients with diastolic orthostatic hypotension were compared with the 718 patients without diastolic orthostatic hypotension (HR 2.039, 95 % C.I. 0.885–4.696, *P* = 0.094) or with the 578 patients who had a normal diastolic blood pressure response (HR 1.818, 95 % C.I. 0.786–4.204, *P* = 0.162). Patients with diastolic orthostatic hypotension had significantly (*P* = 0.013) higher PWV and significantly (*P* = 0.010) higher IMT than patients with a normal orthostatic blood pressure response (Table 2).

### Combined groups

When patients who had either systolic or diastolic orthostatic hypertension or both (*n* = 172) were grouped together and compared with the 534 patients with a normal orthostatic blood pressure response, the presence of any hypertensive orthostatic blood pressure response was associated with a decreased risk for the composite end-point of borderline statistical significance (HR 0.530, 95 % C.I. 0.271–1.036, *P* = 0.064) and with significantly higher PWV (10.8 ± 2.2 m/s vs. 10.2 ± 2.1 m/s, *P* = 0.006) but similar IMT (0.75 ± 0.19 mm vs. 0.73 ± 0.17 mm, *P* = 0.156). When patients who had either systolic or diastolic orthostatic hypotension or both (*n* = 49) were compared with the 534 patients with a normal orthostatic blood pressure response, there was no significant association with the presence of any hypotensive orthostatic blood pressure response and the composite end-point (HR 1.308, 95 % C.I. 0.597–2.866, *P* = 0.502) but significantly higher values were found for both PWV (11.0 ± 2.4 m/s vs. 10.2 ± 2.1 m/s, P = 0.022) and IMT (0.82 ± 0.25 mm vs. 0.73 ± 0.17 mm, *P* = 0.014).

### Sensitivity analysis

Since previous cardiovascular disease is a risk factor for recurrent events, we performed a sensitivity analysis, in which 86 patients were excluded due to reporting either previous myocardial infarction or previous stroke, or due to missing baseline data concerning these variables. When compared with patients with a normal orthostatic blood pressure response, the hazard ratio for the combined end-point was 1.010 (95 % C.I. 0.312–3.272, *P* = 0.986, *n* = 510) for systolic orthostatic hypertension, 1.119 (95 % C.I. 0.270–4.637, P = 0.877, *n* = 493) for systolic orthostatic hypotension, 0.285 (95 % C.I. 0.088–0.923, *P* = 0.036, *n* = 595) for diastolic orthostatic hypertension, and 1.728 (95 % C.I. 0.617–4.837, *P* = 0.298, *n* = 499) for diastolic orthostatic hypotension. For patients with either systolic or diastolic orthostatic hypertension or both, the hazard ratio was 0.394 (95 % C.I. 0.155–0.999, *P* = 0.050, *n* = 624) and for patients with either systolic or diastolic orthostatic hypotension or both, the hazard ratio was 1.342 (95 % C.I. 0.529–3.404, *P* = 0.536, *n* = 516).

## Discussion

The principal finding of this prospective observational cohort study was that diastolic orthostatic hypertension was found in nearly one in five patients with type 2 diabetes, and that this condition was associated with a decreased risk for incident cardiovascular events, independently of classical cardiovascular risk factors.

The prevalence of orthostatic hypertension in our cohort was similar to previously reported data from patients with long term type 1 diabetes [25] and middle- aged [17] or elderly [18] patients with type 2 diabetes. It is difficult, however, to make comparisons between studies, since several different definitions of orthostatic hypertension have been used. In some studies [17, 25], a change from sitting systolic blood pressure < 140 mmHg to standing blood pressure ≥140 mmHg and/or a change from sitting diastolic blood pressure <90 mmHg to standing blood pressure ≥90 mmHg have been used, whereas other authors applied systolic cut-offs of 10 mmHg [18] or 20 mmHg [15, 26]. Diastolic cut-off values of 10 mmHg have been applied in two previous studies only [16, 27], none of which were confined to patients with type 2 diabetes. In the Atherosclerosis Risk in Communities Study [16], a diastolic blood pressure rise >10 mmHg was found in 9.2 % of participants. No association between diastolic orthostatic hypertension and stroke risk was found, but a linear relationship between diastolic orthostatic blood pressure change and incident non-lacunar thrombotic strokes was noted. In the Normative Aging Study [27], which included only presumably healthy men, diastolic blood pressure rise ≥10 mmHg was found in a similar proportion of participants (20.8 %) as in our cohort. The prognostic impact of diastolic orthostatic hypertension was not evaluated, but sitting diastolic blood pressure was a significant risk factor for myocardial infarctions only in patients with an exaggerated diastolic blood pressure rising reaction.

Considering previously reported data from patients with diabetes mellitus [1, 2], we found a lower than expected prevalence of orthostatic hypotensive blood pressure reactions in our cohort. A possible explanation may be that we measured blood pressure in the sitting rather than in the supine position. Although this is in accordance with recent guidelines for resting blood pressure measurements [28], it may have contributed to an underestimation of the prevalence of orthostatic hypotensive reactions. Furthermore, delayed orthostatic hypotension has been found to be quite common in patients undergoing head-up tilt tests [29] and since we measured the standing blood pressure 2 minutes after the patients had risen, patients with later orthostatic hypotension may not have been identified. Indeed, previous studies in which orthostatic hypotension occurred more frequently [1, 2] included systolic measurements obtained after 3 minutes of standing. This discrepancy in methodology may have contributed to the relatively low prevalence of orthostatic hypotension in our cohort. We also did not find any statistically significant associations between orthostatic hypotension and any particular antihypertensive drug class, which is in contrast to other reports, where beta blockers [30] and calcium antagonists and alpha blockers [31] have been associated with this condition.

Since diastolic blood pressure control is particularly important in patients with diabetes mellitus [32], our finding that diastolic orthostatic hypertension was associated with a lower risk for cardiovascular events may at first seem counter-intuitive. However, it is important to bear in mind that the definition of diastolic orthostatic hypertension in our study was based on the change of the diastolic blood pressure, not on absolute diastolic blood pressure levels. In fact, sitting diastolic blood pressure was significantly lower in patients with diastolic orthostatic hypertension, and only a minority of patients with diastolic orthostatic hypertension had standing blood pressure >90 mmHg. The majority of the endpoint events in our trial were related to ischemic heart disease. Since myocardial perfusion occurs mainly during diastole, it could be speculated that an inability to raise the diastolic blood pressure during orthostasis puts the myocardium at increased risk for episodes of hypoperfusion, particularly in the presence of coronary artery disease. Supporting this theory is the finding that in the Captoril Prevention Project, diastolic orthostatic hypotension was a predictor of myocardial infarctions but not of strokes, whereas systolic orthostatic hypotension predicted strokes but not myocardial infarctions [3]. Possibly, this could be a consequence of the cerebral perfusion being more dependent than the myocardial perfusion on systolic blood pressure. The low number of patients with systolic orthostatic hypotension, as well as the relatively low number of strokes that occurred in our cohort, may have precluded us from elucidating any associations between systolic orthostatic hypotension and cerebrovascular disease. It should also be noted that previous investigations have shown an association between a marked blood pressure elevation during exercise stress testing and a good prognosis [33, 34], although the impact of the diastolic blood pressure response has not been studied in detail in these circumstances.

Early pathogenetic studies [35] on orthostatic hypertension showed that in patients with this condition, excessive gravitational pooling of blood in the lower extremities causes a reduction of cardiac output, which leads to a compensatory sympathetic hyperactivity followed by an abnormally intense arterial vasoconstriction which raises the diastolic blood pressure. Increased peripheral vascular resistance has been demonstrated in patients with orthostatic hypertension who underwent a head-up tilt test [36]. Further support of a role for peripheral resistance in the pathogenesis of this condition comes from a case report in which a patient with severe symptomatic orthostatic hypertension was successfully treated with an alpha blocker [37]. We did not perform measurements of endothelial function in our study, but it could be speculated that diastolic orthostatic hypertension is associated with preserved endothelial function, which would help explain the decreased risk associated with this condition in our cohort. In patients with type 2 diabetes, baroreflex hypersensitivity has been suggested as a factor contributing to orthostatic hypertension [17]. Increased arterial stiffness may also be a relevant contributing condition, and we found an association between the presence of systolic orthostatic hypertension and high aortic PWV in our cohort, which is in contrast to what has previously been reported in a smaller pioneering study [38].

In the present study, we did not find a statistically significant increased risk in patients with orthostatic hypotension, regardless of whether diastolic or systolic diagnostic criteria were applied. This is in contrast to several previously published reports [2–12]. This discrepancy may be due simply to a lack of power, since relatively few patients exhibited these blood pressure reactions in our cohort. Therefore, these results should be cautiously interpreted. Furthermore, since we did find an association between diastolic orthostatic hypotension and markers of increased arterial stiffness (PWV) and atherosclerosis (carotid IMT), this condition may not be entirely harmless. Since the value of diastolic rather than systolic orthostatic hypotension as a marker of risk for myocardial infarction has been described previously in three studies [3, 5, 10], our results may suggest that the diastolic orthostatic hypotensive reaction is of particular importance in terms of risk stratification, at least in patients at high risk for coronary artery disease.

Strengths of our study include the community-based approach, in which patients were recruited from primary care with few exclusion criteria and with no patients lost to follow-up. In our opinion, the most important study limitation was that few patients fulfilled the criteria for diastolic or systolic orthostatic hypotension. Thus, the lack of statistically significant associations between orthostatic hypotension and the combined end-point may be attributable to a lack of statistical power. Furthermore, there were relatively few events, which precludes us from exploring possible gender differences, and from separately studying patients with or without antihypertensive medications. Larger studies that directly compare the predictive value of different orthostatic blood pressure reactions, as well as the impact of different antihypertensive drugs on orthostatic blood pressure reactions, should be encouraged in patients with type 2 diabetes. Finally, it should be noted that the orthostatic blood pressure response was diagnosed at one single occasion. Since the reproducibility of orthostatic blood pressure reactions has been shown to be poor [39], it is possible that some patients would have changed orthostatic status if the measurement had been repeated at several occasions. Thus, although diastolic orthostatic hypertension diagnosed at one single occasion was a significant and independent predictor of decreased cardiovascular risk in our cohort, it would seem justified in clinical practice to repeat the measurement on several occasions in order to enhance the prognostic performance.

## Conclusions

Diastolic orthostatic hypertension was common in patients with type 2 diabetes, and was associated with a decreased cardiovascular risk, whereas diastolic orthostatic hypotension was associated with significantly increased degree of sub-clinical target organ damage. Evaluation of the orthostatic blood pressure reaction is easy to perform in everyday clinical praxis, and should be encouraged in patients with type 2 diabetes. We suggest that health care providers pay attention to the diastolic orthostatic blood pressure response, which was frequently abnormal in our cohort, and which may add independent prognostic information beyond traditional risk factors. Larger studies aiming at determining the optimal definitions of orthostatic blood pressure reactions, as well as the number of measurements required to confirm the diagnosis, seem warranted in patients with type 2 diabetes.

## Abbreviations

CARDIPP: cardiovascular risk factors in patients with diabetes—a prospective study in primary care
CI: confidence interval
ECG: electrocardiogram
eGFR: estimated glomerular filtration rate
HbA1c: glycosylated hemoglobinA1
HDL: high-density lipoprotein
HR: hazard ratio
IMT: intima-media thickness
LDL: low-density lipoprotein
PWV: pulse wave velocity
